# Improved Detection of Invasive Pulmonary Aspergillosis Arising during Leukemia Treatment Using a Panel of Host Response Proteins and Fungal Antigens

**DOI:** 10.1371/journal.pone.0143165

**Published:** 2015-11-18

**Authors:** Allan R. Brasier, Yingxin Zhao, Heidi M. Spratt, John E. Wiktorowicz, Hyunsu Ju, L. Joseph Wheat, Lindsey Baden, Susan Stafford, Zheng Wu, Nicolas Issa, Angela M. Caliendo, David W. Denning, Kizhake Soman, Cornelius J. Clancy, M. Hong Nguyen, Michele W. Sugrue, Barbara D. Alexander, John R. Wingard

**Affiliations:** 1 Department of Internal Medicine, University of Texas Medical Branch (UTMB), Galveston, TX, United States of America; 2 Institute for Translational Sciences, UTMB, Galveston, TX, United States of America; 3 Sealy Center for Molecular Medicine, UTMB, Galveston, TX, United States of America; 4 Department of Preventive Medicine and Community Health, UTMB, Galveston, TX, United States of America; 5 Department of Biochemistry and Molecular Biology, UTMB, Galveston, TX, United States of America; 6 MiraVista Laboratories, Indianapolis, IN, United States of America; 7 Harvard University, Boston, MA, United States of America; 8 Biomolecular Resource Facility, UTMB, Galveston, TX, United States of America; 9 Alpert Medical School of Brown University, Providence, RI, USA; 10 University of Manchester, Manchester, United Kingdom; 11 University of Pittsburgh, Pittsburgh, United States of America; 12 University of Florida, Gainesville, FLA, United States of America; 13 Duke University Medical Center, Durham, NC, United States of America; Leibniz Institute for Natural Products Research and Infection Biology- Hans Knoell Institute, GERMANY

## Abstract

Invasive pulmonary aspergillosis (IPA) is an opportunistic fungal infection in patients undergoing chemotherapy for hematological malignancy, hematopoietic stem cell transplant, or other forms of immunosuppression. In this group, *Aspergillus* infections account for the majority of deaths due to mold pathogens. Although early detection is associated with improved outcomes, current diagnostic regimens lack sensitivity and specificity. Patients undergoing chemotherapy, stem cell transplantation and lung transplantation were enrolled in a multi-site prospective observational trial. Proven and probable IPA cases and matched controls were subjected to discovery proteomics analyses using a biofluid analysis platform, fractionating plasma into reproducible protein and peptide pools. From 556 spots identified by 2D gel electrophoresis, 66 differentially expressed post-translationally modified plasma proteins were identified in the leukemic subgroup only. This protein group was rich in complement components, acute-phase reactants and coagulation factors. Low molecular weight peptides corresponding to abundant plasma proteins were identified. A candidate marker panel of host response (9 plasma proteins, 4 peptides), fungal polysaccharides (galactomannan), and cell wall components (β-D glucan) were selected by statistical filtering for patients with leukemia as a primary underlying diagnosis. Quantitative measurements were developed to qualify the differential expression of the candidate host response proteins using selective reaction monitoring mass spectrometry assays, and then applied to a separate cohort of 57 patients with leukemia. In this verification cohort, a machine learning ensemble-based algorithm, generalized pathseeker (GPS) produced a greater case classification accuracy than galactomannan (GM) or host proteins alone. In conclusion, Integration of host response proteins with GM improves the diagnostic detection of probable IPA in patients undergoing treatment for hematologic malignancy. Upon further validation, early detection of probable IPA in leukemia treatment will provide opportunities for earlier interventions and interventional clinical trials.

## Introduction

*Aspergillus* is an important opportunistic fungal pathogen affecting immunocompromised patients, and the disease is associated with high mortality [1, 2]. Invasive aspergillosis is the most common type of fungal infection among stem cell transplant recipients and is the second-most common type of fungal infection among solid organ transplant recipients, with a 12-month cumulative incidence of 19% [3]. Despite intense surveillance and institution of early aggressive anti-fungal therapy, case fatality rates are as high as 50 to 90% depending on underlying disease and site of infection [4, 5].

*Aspergillus spp* are ubiquitous environmental molds whose conidia are easily aerosolized [6]. In the presence of normal innate and adaptive immune systems, airborne fungal spores are usually cleared by resident macrophages that phagocytose and destroy them [7]. In patients with an intact immune response, *Aspergillus* is responsible for a spectrum of diseases ranging from aspergilloma, allergic sinusitis or bronchopulmonary aspergillosis, to chronic necrotizing pulmonary aspergillosis. By contrast, in patients with suppressed immunity such as those undergoing hematopoietic stem cell transplant, organ transplant, or those undergoing induction therapy for hematological malignancy, *Aspergillus* can cause aggressive and invasive infection leading to devastating outcome [6]. Specifically, in patients with acute leukemia, prolonged periods of neutropenia and dysfunctional macrophages are major risk factors for invasive pulmonary aspergillosis [IPA; ref [8]].

Within the immunocompromised airway, colonizing conidia germinate into a replicative and invasive hyphal form, producing angioinvasion, inflammation, and hematogenous fungal spread. In the invasive stages, the fungus disseminates via the blood to involve multiple organ systems including the liver and central nervous system [6]. Because of its protean manifestations, IPA is difficult to diagnose early in the course of infection. Clinically, diagnosis of IPA is established on the basis of radiographic, culture, and fungal antigen detection in high-risk patients [6, 9]. Despite hematogenous dissemination, fungal blood cultures are rarely positive [10]. The development of fungal antigen assays has added to the available diagnostic tools. Here, clinical grade ELISA assays for galactomannan (GM), a polysaccharide produced during hyphal growth [11], and (1, 3)-β-D-glucan (BG), a fungal cell wall component, have been developed [12]. Although GM and BG measurement in blood are used clinically for the detection of opportunistic fungal diseases, these tests are subject to false positive and false negative effects, thereby limiting their clinical usefulness [11, 13]. More sensitive and accurate diagnostic panels will have impact on the management and morbidity from IPA.

Because prompt diagnosis of IPA improves survival [14, 15], we sought to discover, qualify, and verify a panel of protein biomarkers associated with proven and probable IPA using plasma specimens from the *Aspergillosis* Technology Consortium (AsTeC) and proteomics analysis performed by the UTMB Clinical Proteomics Center (CPC). We hypothesized that the inclusion of host response proteins to that of fungal antigens would improve the early detection of IPA.

## Materials and Methods

### Ethical approval

This study was conducted by the Aspergillus Technology Consortium (AsTeC) under approval by the University of Florida Institutional Review Board (IRB) protocol 073–2008 and by the UTMB Clinical Proteomics Center (CPC) under approval from UTMB IRB No. 08–258. This study was compliant with all applicable federal regulations governing the protection of human subjects. Most subjects were adults who provided written, informed consent prior to participation in the study. Subjects under the age of 18 provided either written informed consent or assent with parent/guardian providing written consent.

### Study design

Three patient populations at high risk for IPA were enrolled by informed consent or assent at the start of their transplant or chemotherapy treatment at the 3 AsTeC collection sites. These included subjects undergoing hematopoietic stem cell transplant, subjects receiving intensive chemotherapy for hematologic malignancies, and subjects receiving lung transplants. Each patient was scored for IPA status (proven, probable or no IPA) using case definitions established by the Mycoses Study Group / European Organization for Research and Treatment of Cancer (MSG/EORTC) [16, 17].

All cases included in this study met the 2008 revised EORTC/MSG criteria of proven or probable IPA ([16]; diagnostic criteria are shown in S1 Table). Of the 61 IPA cases, serum GM was assayed in 60- of which 36 tests were positive (S2 Table). Bronchoalveolar lavage GM was assayed in 35 cases- of which 30 were positive. A positive serum GM was the only mycological criteria in 22 of the 61 cases. Independent testing of GM was performed on all IPA cases using serum collected on the same day as samples used for proteomics analysis. Those who developed probable or proven IPA where selected for subsequent proteomics studies. The median time between IPA case sample collection and EORTC date was 0 days (range 67 days before EORTC to 38 days after EORTC).

Blood samples were also available from 17 cases prior to their diagnosis of probable IPA, termed “auto-controls”. The median days between auto control sample collection and EORTC date was 28 days before EORTC (range 10–390 days). Cases with other concomitant fungal infections were excluded. No invasive fungal infections were identified in the control group. Bacterial sepsis was present in 3 of the controls within 7 days before or after the sample used for testing in this study. Controls without IPA were selected from the same cohort, and matched for gender, age and recruiting center.

**Fig 1:**
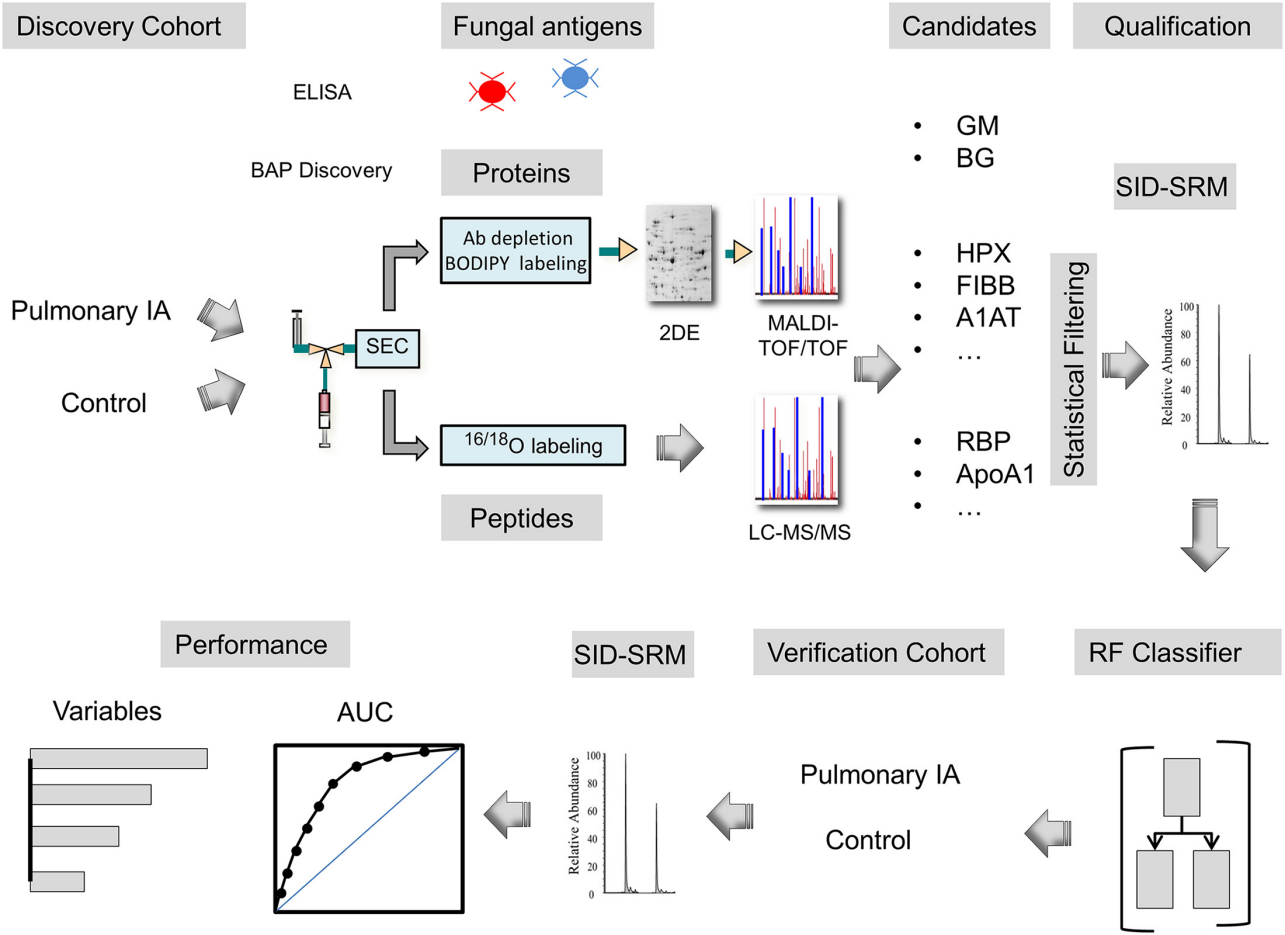
Schematic view of panel development for pulmonary IPA. Schematic view of strategy for discovery, qualification, and verification of panel-based classifiers. The BAP fractionation platform fractionates proteins and peptides for analysis by automated size exclusion chromatography (SEC). Candidate biomarkers were assembled based on proteins identified in the discovery phase and by previous studies. For each candidate, targeted proteomics assays using stable isotope dilution (SID)-selected reaction monitoring (SRM) assays were developed, standardized, and used to quantitate the abundance of the candidate biomarker in the discovery population (qualification). Nonparametric statistical filters were used to identify 15 host response proteins/peptides and 2 fungal polysaccharides. SID-SRM-MS measurement of host-response proteins and peptides were used to test RF classifier performance.

Because no host response proteins have yet been discovered clinically for the diagnosis of IPA, we undertook a broad discovery approach for discriminant features in plasma using targeted immunoassays, as well as discovery proteomics and analysis of discriminant peptides using the biofluids analysis platform (BAP, schematically diagrammed in Fig 1).

### Sample QA/QC and analysis

Blood was collected in a closed system Vacutainer^®^ CPT^™^ Cell Preparation Tube anti-coagulated with sodium citrate (Becton, Dickinson and Company, Franklin Lakes, New Jersey). The plasma fraction was separated from cells and frozen at -80°C using a standard operating procedure implemented across all study sites. Blood was also collected in BD Vacutainer^®^ Plus plastic serum tubes (Becton, Dickinson and Company, Franklin Lakes, New Jersey, separated by centrifugation and serum frozen at -80°C for GM and BG testing. All samples and clinical data were de-identified and linked to an anonymous study ID number at the AsTeC Biorepository. For proteomics assays, each plasma sample was assayed for protein content, and a few randomly selected for cysteine content estimation by amino acid analysis (for quantitative cysteine-specific fluorescence-labeling). Plasma protein profiles were determined for all samples by capillary electrophoresis. For the 2D gel electrophoresis (2DE) and peptide analyses, specimens were randomized for proteomics analysis.

### Biofluid Analysis Platform (BAP) Fractionation and 2DE

The BAP pre-separation fractionation system is a semi-automated device that fractionates denatured plasma into protein and peptide pools as described earlier in detail [18]. The protein pools were depleted of the 14 most highly abundant proteins and labeled with BODIPY FL-maleimide (BD) [19, 20]. BD-labeled proteins were separated by 2DE, imaged, and analyzed using SameSpots software (Totallab, Ltd. Newcastle Upon Tyne, UK). The peptide pools were digested and stable isotope labeled using trypsin digestion in the presence of H_2_^18^O, described in detail below.

### Protein Identification

2DE spots that were significantly differentially expressed were picked robotically, trypsin-digested, and peptides identified by MALDI TOF/TOF (AB Sciex 5800, Foster City, CA). Data were analyzed with the AB Sciex software package included with the instrument: TOF/TOF Series Explorer (v. 4.1.0) with Oracle Database Schema (v. 4.0.0), and Data Version (v. 4.0.5) to acquire both MS and MS/MS spectral data, respectively. The instrument was operated in positive ion reflectron mode, mass range was 850–3000 Da, and the focus mass was set at 1700 Da. For MS data, 600–1000 laser shots were acquired and averaged from each sample spot. Automatic external calibration was performed using a peptide mixture with reference masses 904.468, 1296.685, 1570.677, and 2465.199.

Following MALDI MS analysis, MALDI MS/MS was performed on several (5–10) abundant ions from each sample spot. A 1kV positive ion MS/MS method was used to acquire data under post-source decay (PSD) conditions. The instrument precursor selection window was +/- 3 Da. For MS/MS data, 1500 laser shots were acquired and averaged from each sample spot. Automatic external calibration was performed using reference fragment masses 175.120, 480.257, 684.347, 1056.475, and 1441.635 (from precursor mass 1570.700).

AB Sciex ProteinPilot software was used in conjunction with MASCOT to search the respective protein database using both MS and MS/MS spectral data for protein identification. Protein match probabilities were determined using expectation values and/or MASCOT protein scores. MS peak filtering included the following parameters: mass range 850 Da to 3000 Da, minimum S/N filter = 10, mass exclusion list tolerance = 0.5 Da, and mass exclusion list (for some trypsin and keratin-containing compounds) included masses 842.51, 870.45, 1045.56, 1179.60, 1277.71, 1475.79, and 2211.1. For MS/MS peak filtering, the minimum S/N filter = 10. For protein identification, the *Homo sapiens* taxonomy was searched in the NCBI database. Other parameters included the following: trypsin proteolysis; 1 maximum missed cleavage; fixed modifications included carbamidomethyl (C) for 2DE analyses only; variable modifications included oxidation (M); precursor tolerance was set at 0.5 Da; MS/MS fragment tolerance was set at 0.5 Da; mass = monoisotopic; and peptide charges were only considered as +1. The entire protein list was also searched for the *Aspergillus* database (http://www.aspergillusgenome.org). Species searched included *Aspergillus clavatus* NRRL 1; *Aspergillus flavus* NRRL 3357; *Aspergillus fumigatus* A1163, Af293; *Aspergillus nidulans* FGSC A4; *Aspergillus niger* ATCC 1015, CBS 513.88; *Aspergillus oryzae* RIB40/ATCC 42149; *Aspergillus terreus* NIH2624 and *Neosartorya fischeri* NRRL 181.

### Peptide analysis

The peptide pools obtained from the BAP were differentially labeled with ^16/18^O by trypsin-mediated exchange. To ensure maximum incorporation of two oxygen isotopes at each carboxy-terminus, the catalytic incorporation of the first oxygen is performed at pH 8.0 and the second non-catalytic exchange is performed at pH 6.0 for 24 h. Incorporation is monitored by triple quadrupole mass spectrometry. Labeled peptides were then separated by RP-HPLC, and quantified and identified by a tandem electrospray MS/MS (AB 4000 Q-trap). 18 peptides were detected in greater than 50% of the sample set. These were developed into independent SID-SRM assays for exact measurement and assessment for differential expression. Of these, 3 were statistically significant between cases and controls.

### Multiplex ELISA

Plasma concentrations of interleukin (IL)-6, -10, tumor necrosis factor (TNF)-α, plasminogen activator inhibitor (PAI)-1, Factor VIII, and Von Willebrands’s factor (vWF) were assayed by sandwich ELISA (Bioplex, Bio-Rad, Hercules, CA) as previously described [18]. Data were log2 normalized for stastistical analysis and modeling.

### Stable Isotope Dilution (SID)-Selected Reaction Monitoring (SRM)-MS

Signature peptides with their native flanking sequences around the trypsin cleavage site were chemically synthesized incorporating isotopically labeled [^13^C_6_^15^N_4_] arginine or [^13^C_6_^15^N_2_] lysine to a 99% isotopic enrichment (Thermo Scientific). For intact protein analysis, 5 μL of plasma samples were denatured with 145 μL of 8M urea. Known amounts of stable isotope labeled peptide standards were spiked into each sample. After reduction/alkylation with DTT and iodoacetamide, the plasma samples were digested with trypsin. The peptides were desalted with C18-ZipTip (Millipore, MA) prior to LC-MS analysis. For plasma peptides collection, 3 mg of plasma protein, 8 M urea, and 3 μg of purified Alexa-488 labeled thaumatin (Sigma-Aldrich, St. Louis, MO) in a final volume of 300 μL, were fractionated into “peptide pools” by SEC in BAP. Known amount of stable isotope labeled standard peptides were added into the peptide pool and digested with trypsin. The tryptic peptides were desalted with SepPak C18 cartridge (Waters, MA), and subjected to SID-SRM.

LC-SRM-MS analysis was performed with a TSQ Vantage triple quadrupole mass spectrometer equipped with nanospray source (Thermo Scientific, San Jose, CA). The online chromatography were performed using an Eksigent NanoLC-2D HPLC system (AB SCIEX, Dublin, CA). An aliquot of 10 μL of each of tryptic digests were injected on a C18 reverse-phase nano-HPLC column (PicoFrit^™^, 75 μm x 10 cm; tip ID 15 μm) at a flow rate of 500 nL/min with a 20-min 98% A, followed by a 15-min linear gradient from 2–30% mobile phase B (0.1% formic acid-90% acetonitrile) in mobile phase A (0.1% formic acid). The TSQ Vantage was operated in high-resolution SRM mode with Q1 and Q3 set to 0.4 and 0.7-Da Full Width Half Maximum (FWHM). All acquisition methods used the following parameters: 2100 V ion spray voltage, a 275°C ion transferring tube temperature, a collision-activated dissociation pressure at 1.5 mTorr, and the S-lens voltage used the values in S-lens table generated during MS calibration.

All SRM data were manually inspected to ensure peak detection and accurate integration. The chromatographic retention time and the relative product ion intensities of the analyte peptides were compared to those of the stable isotope labeled standard (SIS) peptides. The variation of the retention time between the analyte peptides and their SIS counterparts should be within 0.05 min, and the difference in the relative product ion intensities of the analyte peptides and SIS peptides were below 20%. The peak area in the extract ion chromatography of the native and SIS version of each signature peptide were integrated using Xcalibur^®^ 2.1. The default values for noise percentage and base-line subtraction window were used. The ratio between the peak area of native and SIS version of each peptide were calculated. Two technical replicates of analysis for each sample were performed and each technical replicate was analyzed by SRM-MS twice. Within-technical replicates were used to assess assay reproducibility.

### Galactomannan (GM) assay

The Platelia *Aspergillus* enzyme immunoassay (EIA) (Bio-Rad Laboratories, Redmond, WA) was performed according to the manufacturer’s procedures at MiraVista Laboratories (Indianapolis, IN, Ref [13]). Results were expressed as GM indices defined as the ratio of the optical density (OD) value of the sample to the OD value of a standard sample containing 1 ng of GM.

Independent testing of GM was performed on all IPA cases using serum collected on the same day as samples used for proteomics analysis. The median number of days between clinical diagnostic serum GM and independent testing was 3.5 days (range 41 days before clinical diagnostic to 57 days after clinical diagnostic). GM and independently tested serum GM were the same (positive or negative) in 44 of the 60 cases tested (S2 Table). Of the 14 cases where clinical GM and study GM differed, all clinical diagnostic GM tests were positive and independently tested GM were negative. In addition, of these 14 cases, 13 were undergoing antifungal therapy at the time of collection of the independent tested GM sample, and in 1 case, the sample was collected 4 days after antifungal therapy concluded. Only serum was used for GM analysis. The cut off for determination of positive GM was GM ≥ 0.5.

### (1, 3)-beta-D-glucan (BG) assay

BG testing was performed at MiraVista Diagnostics (Indianapolis, IN) using the Fungitell assay (Associates of Cape Cod Inc., East Falmouth, MA), in accordance with the manufacturer's instructions [21]. For the purposes of our study, specimens were tested in duplicate and BG levels were reported numerically (12).

### Statistical analysis and model

Statistical comparisons were performed using SPSSv20 (SPSS, Inc., Chicago, IL). 2-way ANOVA, Mann-Whitney, chi-squared and Kruskall Wallis tests were applied as indicated. One way ANOVA was performed on the log2 transformed spot volumes. The significance level in all instances was 0.05. Random Forests [22, 23], CART, and multivariate regression spline (MARS) modeling was performed by 10-fold cross validation using Salford Predictive Modeler (v7.0, Salford Systems Inc). Data were z-scored prior to model building. Measurements from the discovery cohort were used as the “training” data set, and those from the verification cohort as the “test” data set. Model performance was evaluated by analysis of the AUC of the ROC curve, where sensitivity (true positive) vs 1-specificity (false positive) was plotted and assessment of classification accuracy [22]. Modeling in Random Forests assigned weights to outcomes with case = 1 and control = 0.5 in order to obtain better performance in predicting cases.

## Results

The participants in the discovery cohort were composed of 46 unique men and 22 unique women, with a mean age of 52.66 ± 13.59 years. Forty six patients had leukemia as an underlying disease with 22 other (lung- and hematopoietic stem cell transplants). In 17 cases, blood samples were available from the surveillance study that were taken prior to the diagnosis of probable IPA; these samples termed “auto” controls were also included in the discovery analysis to minimize th effects of individual variations in the plasma proteome on the biomarker identification. There was no statistical difference in the ages, gender distribution, primary diagnosis, center collection site or neutrophil count between the case and control groups (Table 1). Patients with hematological malignancies underwent intensive chemotherapy producing prolonged neutropenia. The distribution of treatment type for cases and controls is shown in Table 2.

**Table 1:** Characteristics of study cohorts.

Cohort	Disease	Age, years (mean ± S.D.)	GenderNo. (M/F)	Underlying Disease, number (Leukemia/Other)	Absolute Neutrophil Count (billion cells/L) (Median (IQR))
**Discovery (N = 85)**					
	ControlN = 34	48.35 ± 14.79 yr	20/14	25/9	1.3 (0.3, 3.9)
	AutoN = 17	54.17 ± 11.27 yr	10/7	9/8	1.3 (0.4, 3)
	CaseN = 34	56.97 ± 10.87 yr	20/14	21/13	0.3 (0, 1.8)
	Total SamplesN = 85	52.96 ± 13.10 yr	50/35	55/30	1.0 (0.1, 3.1)
	Total Unique IndividualsN = 68	52.66 ± 13.59 yr	40/28	46/22	0.9 (0.1, 3.0)
**Qualification (N = 37)**					
	ControlN = 20	49.2 ± 16.68 yr	13/7	20/0	2.5 (0.7, 5.3)
	CaseN = 17	57.12 ± 12.18 yr	10/7	17/0	0.0 (0.0, 0.8)
	TotalN = 37	52.84 ± 15.12 yr	23/14	37/0	1.1 (0.0, 3.1)
**Verification (N = 57)**					
	ControlN = 30	50.27 ± 15.17 yr	23/7	30/0	1.5 (0.1, 3.7)
	CaseN = 27	55.07 ± 13.31 yr	20/7	27/0	0.1 (0.0, 1.7)
	TotalN = 57	52.54 ± 14.4 yr	43/14	57/0	0.4 (0.0, 3.0)

**Table 2:** Treatment regimens for case and controls, by diagnosis.

TREATMENT CATEGORY	IPA CASES	MATCHED CONTROLS
**BMT**	22	29
**Allo**	21	29
**Auto**	1	0
**Hem**. **Malig**.	37	34
**AML**	24	28
**CLL**	5	1
**MDS**	3	1
**NHL**	2	1
**ALL**	1	2
**CML**	1	1
**CMML**	1	0
**Lung Transplant (COPD)**	1	1

Because previous studies demonstrated increased circulating levels of cytokines endothelial-derived proteins in patients with chronic necrotizing pulmonary aspergillosis [24]., we measured IL-6 and -10, tumor necrosis factor (TNF)- α, PAI-1, Factor VIII, and vWF. Of these measurements, only IL-6 was significantly elevated in case over that of control (p<0.05, Wilcoxon Ranked Sum Test, S3 Table and S1 Fig).

We next applied discovery proteomics to identify differentially abundant proteins using an unbiased protein profiling strategy (BAP; Methods and Fig 1). Five hundred fifty-six spots were detected by 2DE; of these, 66 were identified to be statistically significant across the groups (Table 3). High confidence identifications of anti-proteases, acute-phase inducible products of the complement and coagulation cascades, and apolipoproteins were found. Interestingly, multiple identifications of the same protein were observed as both different isoelectric forms and apparent molecular weight. For example, fibrinogen beta (FIBB) was observed at isoelectric points (pIs) of 7.66 and 5.99 (spots 1, 2 in Table 3) and hemopexin (HPX) was observed at pIs of 5.02 and 6.35 (spots 17, 18 in Table 3). FIBA was observed at two distinct molecular weights of 19 kDa and 12 kDa (spots 11 and 13 in Table 3). These observations indicate active enzymatic modification or proteolysis, respectively by IPA.

**Table 3:** Protein Identification.

NO.	PROTEIN NAME	ACC NO	PI	MW	MS EXPEC. VALUE	P-VALUE	ABUNDANCE RATIO (CASE VS CONTROL)
**1**	Fibrinogen beta chain	P02675	7.66	51	1.26E-16	0.030148	1.16
**2**	Fibrinogen beta chain	P02675	5.99	49	3.97E-14	0.049690	1.14
**3**	Alpha-mannosidase 2	Q16706	7.51	36	1.99E+01	0.003111	-1.41
**4**	Ig kappa chain V-III region	P01620	8.19	35	9.98E-12	0.000343*	-1.54
**5**	Ig kappa chain V-III region	P01620	7.15	35	1.58E-04	0.000673*	-1.82
**6**	Ferritin light chain	P02792	7.04	31	3.97E-13	0.000492*	2.12
**7**	Leucine-rich alpha-2-glycoprotein	P02750	8.12	17	1.58E-24	0.001784	1.71
**8**	Complement factor B	P00751	7.31	73	7.92E-09	0.037614	-1.31
**9**	Hemopexin HPX	P02790	7.38	31	6.29E-03	0.005740	-1.36
**10**	Serum amyloid A-4 protein	P35542	3.76	25	5.00E-01	0.001691	-1.44
**11**	Fibrinogen alpha chain	P02671	5.37	19	6.29E-37	0.031877	1.25
**12**	Leucine-rich alpha-2-glycoprotein	P02750	8.21	17	1.26E-26	0.002389	1.43
**13**	Fibrinogen alpha chain	P02671	3.96	12	1.99E-14	0.005660	-1.37
**14**	Complement C3	P01024	5.60	48	1.58E-30	0.005304	-1.31
**15**	Complement C4-A	P0C0L4	7.25	42	1.99E+00	0.013122	-1.35
**16**	Histidine protein methyltransferase 1 homolog METTL18	O95568	7.55	31	3.97E+01	0.043937	-2.32
**17**	Hemopexin HPX	P02790	5.22	30	2.51E-04	0.010053	-1.28
**18**	Hemopexin HPX	P02790	6.35	30	1.58E+01	0.037373	-1.28
**19**	Keratin, type II cytoskeletal 1	P04264	9.26	29	3.15E-03	0.043690	-1.12
**20**	Serum amyloid A-4 protein	P35542	6.68	26	1.58E-05	0.047502	-1.31
**21**	MEF2-activating motif and SAP domain-containing transcriptional regulator	Q6ZN01	8.10	20	2.51E+01	0.046841	-1.58
**22**	Apolipoprotein A-II	P02652	7.03	20	6.29E-05	0.021182	-1.36
**23**	Alpha-1-antichymotrypsin	P01011	5.00	19	1.99E-60	0.005314	1.38
**24**	Alpha-1-antichymotrypsin	P01011	7.74	19	9.98E-60	0.020735	1.33
**25**	Alpha-1-antichymotrypsin	P01011	5.51	19	3.15E-19	0.004400	1.60
**26**	Alpha-1-antitrypsin	P01009	4.87	17	9.98E-01	0.003011	1.33
**27**	Leucine-rich alpha-2-glycoprotein	P02750	6.33	17	6.29E-34	0.008686	1.29
**28**	Leucine-rich alpha-2-glycoprotein	P02750	9.18	17	2.51E-36	0.009977	1.37
**29**	Alpha-1-antitrypsin	P01009	3.85	16	1.26E-12	0.031016	1.29
**30**	Alpha-1-antitrypsin	P01009	4.81	16	1.26E-23	0.042439	1.37
**31**	Alpha-1-acid glycoprotein 1	P02763	5.05	14	1.58E-05	0.001129	1.54
**32**	Alpha-1-acid glycoprotein 1	P02763	7.82	14	1.58E-01	0.003841	1.51
**33**	Apolipoprotein A-I	P02647	8.83	13	2.51E-20	0.024695	-1.35
**34**	Apolipoprotein A-I	P02647	7.59	13	3.97E-06	0.006216	-1.44
**35**	Apolipoprotein A-I	P02647	6.74	12	3.15E-33	0.021568	-1.33
**36**	Alpha-1-acid glycoprotein 1	P02763	4.81	12	9.98E-08	0.001122	1.51
**37**	Serum albumin	P02768	6.70	35	3.97E-06	0.022091	1.26
**38**	Alpha-1-antichymotrypsin	P01011	5.60	19	3.97E-55	0.036387	1.35
**39**	Alpha-1-antichymotrypsin	P01011	5.75	19	1.58E-48	0.041962	1.42
**40**	Alpha-1-antichymotrypsin	P01011	6.14	19	1.26E-48	0.006482	1.43
**41**	Alpha-1-antichymotrypsin	P01011	5.25	19	9.98E-47	0.004255	1.50
**42**	Leucine-rich alpha-2-glycoprotein	P02750	8.99	17	2.51E-32	0.002945	1.35
**43**	Leucine-rich alpha-2-glycoprotein	P02750	9.09	17	3.97E-30	0.004495	1.33
**44**	Leucine-rich alpha-2-glycoprotein	P02750	5.90	17	3.97E-34	0.004792	1.31
**45**	Alpha-1-antitrypsin	P01009	3.52	16	9.98E-40	0.045945	1.33
**46**	Alpha-1-antitrypsin	P01009	8.16	16	3.15E-33	0.000936	1.48
**47**	Alpha-1-acid glycoprotein 1	P02763	4.57	14	3.15E-23	0.014537	1.51
**48**	Alpha-1-acid glycoprotein 1	P02763	3.55	14	3.97E-19	0.005862	1.48
**49**	Alpha-1-acid glycoprotein 1	P02763	3.99	12	9.98E-21	0.002073	1.45
**50**	Alpha-1-acid glycoprotein 1	P02763	4.64	12	3.15E-19	0.021949	1.39
**51**	12345 Serum albumin	P02768	9.47	109	6.29E-42	0.027907	-1.12
**52**	Serum albumin	P02768	8.14	73	3.97E-17	0.000148*	-1.62
**53**	Serum albumin	P02768	8.20	73	6.29E-35	0.003984	-1.39
**54**	Complement C4-A	P0C0L4	6.22	73	3.97E-12	0.035901	-1.56
**55**	Fibrinogen alpha chain	P02671	6.22	12	1.99E-14	0.045355	-1.42
**56**	Fibrinogen beta chain	P02675	5.52	51	9.98E-27	0.001817	1.29
**57**	Fibrinogen beta chain	P02675	5.60	51	9.98E-37	0.007696	1.27
**58**	Putative uncharacterized protein C6orf50 C6orf50	Q9HD87	6.25	26	3.15E+01	0.001200	-1.76
**59**	Transthyretin	P02766	6.95	26	1.99E+00	0.001374	-1.48
**60**	Transthyretin	P02766	4.00	26	1.58E-19	0.000343*	-1.50
**61**	Transthyretin	P02766	5.35	26	2.51E-20	0.000116*	-1.62
**62**	Apolipoprotein C-III	P02656	5.36	20	3.15E-11	0.013668	-1.63
**63**	Zinc-alpha-2-glycoprotein	P25311	7.65	17	1.99E-06	0.037615	1.25
**64**	Histidine protein methyltransferase 1 homolog METTL18	O95568	5.56	12	1.99E+01	0.022806	-1.39
**65**	Annexin A10	Q9UJ72	6.84	15	2.51E+01	0.000098*	-2.21
**66**	Transthyretin	P02766	6.76	14	1.26E+00	0.000011*	-1.97

Protein identification was performed using a Bayesian algorithm where matches were characterized by an expectation score, which represents an estimate of the number of matches that would be expected in that database if the matches were completely random. Significance was determined by one-way ANOVA using log2 transformed spot volumes.

*, significantly different after Benjamini-Hochburg correction for multiple hypothesis testing.

These values are for spot 4, p = 0.038105; spot 5, p = 0.046795; spot 6, p = 0.039113; spot 52, p = 0.020548; spot 60, p = 0.031786; spot 61, p = 0.021551; spot 65, p = 0.027383; and spot 66 p = 0.005949.

**Fig 2:**
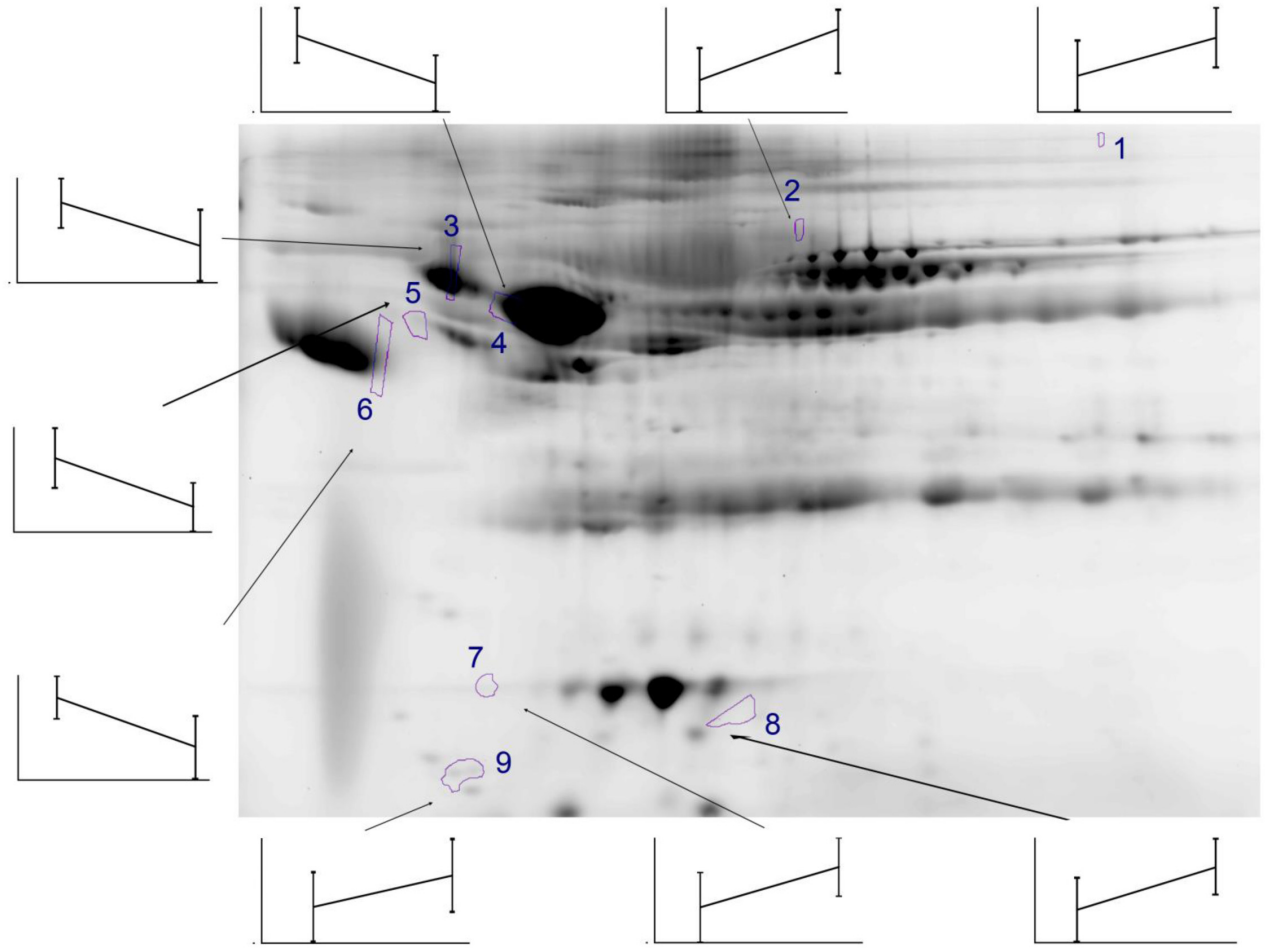
Reference gel of plasma proteins dysregulated by IPA. Shown is a reference gel of 2DE of SEC fractionated and IgY depleted plasma proteins from the study subjects. The location of 9 spots, identified as candidate discriminant proteins are shown. Spot #1 is FIBB; #2 is ALBU; #3 is AATL; #4 is A1AT; #5 is LRG1; #6 is A1AG1; #7 is APO A1; #8 is FIBA; and #9 is APO C3. The image shown represents the pI range of 3–10 and the molecular size range of 25–200+ kDa. Inset, average abundance of each protein spot is shown graphically for Case (Left) and Control (Right) as shown for protein spot # 1.

Genome ontology enrichment analysis showed significant overrepresentation of complement cascade (5-fold enrichment, p < 1.55 X 10^−2^). Consistent with the lack of circulating organisms in patients with acute IPA [10], no high confidence protein matches were obtained by searching peptide fingerprints within multiple *Aspergillus* databases (Materials and Methods). From these data we concluded that complement and coagulation cascades were components of the host response to IPA, and that active post-translational modification of these proteins were produced, perhaps through their consumption or proteolysis as a consequence of angio-invasion. Quantitative SID-SRM-MS analyses were developed for candidate plasma proteins (Fig 2, Table 4).

**Table 4:** SID-SRM-MS assays for candidate plasma proteins.

Protein Name	Accession #	Gene Name	Sequence	Q1 m/z	Q3 m/z	CE (V)	Pre Z	Prod Z	Ion type
**Serum albumin**	P02768	ALBU	LVNEVTEFAK	575.3111	694.3765	23	2	1	y6
				575.3111	823.4191	23	2	1	y7
				575.3111	937.462	23	2	1	y8
				575.3111	1036.53	23	2	1	y9
**Apolipo-protein A-I**	P02647	APOA1	DYVSQFEGSALGK	700.8382	808.4194	27	2	1	y8
				700.8382	936.478	27	2	1	y9
				700.8382	1023.51	27	2	1	y10
				700.8382	1122.578	26	2	1	y11
**Alpha-1-antichymotrypsin**	P01011	AATC	EIGELYLPK	531.2975	633.3965	21	2	1	y5
				531.2975	819.4605	21	2	1	y7
				531.2975	762.4391	21	2	1	y6
				531.2975	932.5446	21	2	1	y8
**Leucine-rich alpha-2-glycoprotein**	P02750	LRG1	GQTLLAVAK	450.7792	501.339	19	2	1	y5
				450.7792	614.423	19	2	1	y6
				450.7792	715.4707	19	2	1	y7
				450.7792	843.5293	19	2	1	y8
**Alpha-1-antitrypsin**	P01009	A1AT	SVLGQLGITK	508.3109	659.4081	21	2	1	y6
				508.3109	716.4296	21	2	1	y7
				508.3109	829.5136	21	2	1	y8
				508.3109	928.582	21	2	1	y9
**Apolipo-protein C-III**	P02656	APOC3	DALSSVQESQVAQQAR	858.929	887.469	33	2	1	y8
				858.929	1016.511	33	2	1	y9
				858.929	1144.57	33	2	1	y10
				858.929	1243.638	33	2	1	y11
**Fibrinogen beta chain**	P02675	FIBB	SILENLR	422.748	531.288	18	2	1	y4
				422.748	644.372	18	2	1	y5
				422.748	757.456	18	2	1	y6
				422.748	844.488	18	2	1	y7
**Alpha-1-acid glycoprotein 1**	P02763	A1AG1	YVGGQEHFAHLLILR	876.9808	982.6191	33	2	1	y8
				876.9808	1119.678	32	2	1	y9
				876.9808	1248.721	31	2	1	y10
				876.9808	835.5507	33	2	1	y7
**Fibrinogen alpha chain**	P02671	FIBA	GLIDEVNQDFTNR	760.871	780.363	29	2	1	y6
				760.871	894.406	29	2	1	y7
				760.871	993.474	29	2	1	y8
				760.871	1122.517	29	2	1	y9
				760.871	1237.544	29	2	1	y10

For each of the candidate plasma proteins, SID-SRM-MS assays were developed. Shown is the protein accession number, common name, signature sequence, quadrupole (Q) mass, optimized collision enegery (CE) and ion type measured. Pre, precursor. Prod, product.

Based on the observation that proteolytic fragments of complement, acute-phase, and coagulation factors were detected in IPA cases, and the knowledge that angio-invasion is locally tissue destructive, we focused biomarker identification on the peptide pools. To accomplish this, we designed quantitative SID-SRM-MS for the 18 peptides represented in >50% of samples. Four peptides were significantly different; these assays are shown in Table 5.

**Table 5:** SID-SRM-MS assays for BAP peptides.

Protein Name	Access #	Gene Name_PreZ	Sequence	Q1 m/z	Q3 m/z	CE (V)	Pre Z	Prod Z	Ion type
Retinol binding protein 4	Q5VY30	RBP4_599	YWGVASFLQK	599.8163	622.3553	24	2	1	y5
				599.8163	693.3925	24	2	1	y6
				599.8163	792.4609	24	2	1	y7
Apolipoprotein A-II	P02652	APOA2_600	VKSPELQAEAK	600.3351	659.3717	24	2	1	y6
				600.3351	788.4143	24	2	1	y7
				600.3351	885.4671	24	2	1	y8
				600.3351	972.4991	24	2	1	y9
Apolipoprotein A-II	P02652	APOA2_486	SPELQAEAK	486.7534	546.2877	20	2	1	y5
				486.7534	659.3717	20	2	1	y6
				486.7534	788.4143	20	2	1	y7
Apolipoprotein A-II	P02652	APOA2_578	SKEQLTPLIK	578.8504	684.4649	23	2	1	y6
				578.8504	812.5234	23	2	1	y7
				578.8504	941.566	23	2	1	y8

For each of candidate peptide purified by SEC in BAP, SID-SRM-MS assays were developed. Shown is the protein accession number, common name, signature sequence, quadrupole (Q) mass, optimized collision enegery (CE) and ion type measured.

We subjected the discovery data to subgroup analysis. Although there was no difference by gender, the 8 candidate plasma proteins, 4 peptides, BG, and GM were different in cases *vs* controls of patients with primary underlying diagnosis of leukemia. None of these candidates were different between cases *vs* controls for other primary diagnoses (e.g., hematopoietic stem cell- or lung transplant). We therefore focused on development of a biomarker panel only for patients with the primary underlying diagnosis of leukemia.

### Assembly and qualification of a candidate biomarker panel for IPA in patients with primary diagnosis of leukemia

**Fig 3:**
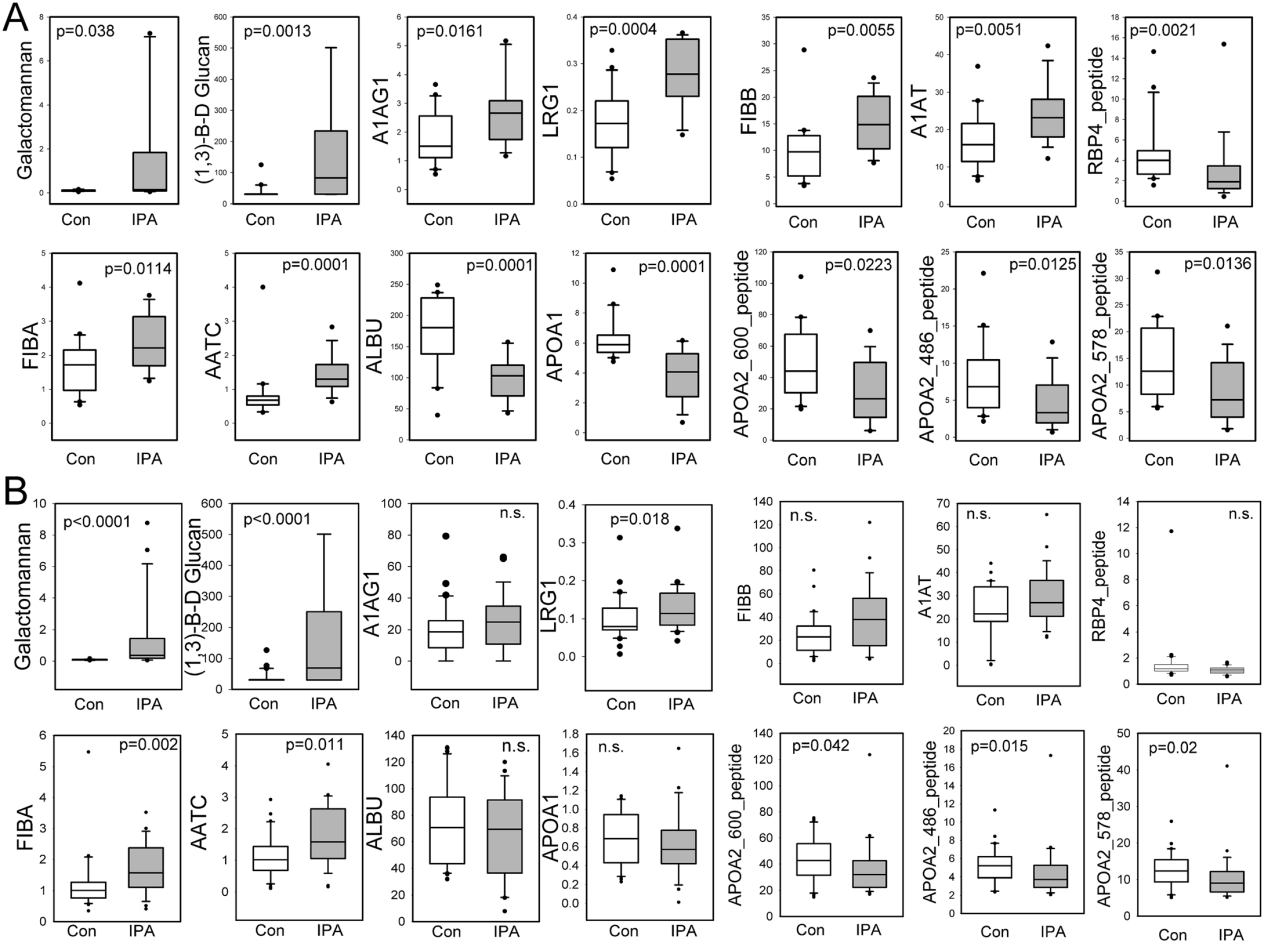
SID-SRM-MS measurements of candidate biomarkers in IPA. A). SID-SRM-MS measurements of the discovery cohort. For each candidate biomarker, the SID-SRM-MS measurements by disease category are shown. Box plots show the 25% and 75% interquartile range and the median value, indicated by horizontal dark line. Outliers are signified with circles. Note that the median horizontal line is not symmetrically located within the box plot, signaling that the data are not normally distributed. P values are from Wilcoxon ranked sum. B). SID-SRM-MS of the validation cohort. Data are presented as in Panel A.

We then developed highly selective SID-SRM-MS assays for the most informative 8 candidate proteins and 4 peptides and independently measured these in the leukemic qualification cohort (Table 1) to confirm their differential expression. Assay characteristics are shown in Tables 4 and 5. These results showed that the measurement of fungal antigens by ELISA and those of the host response proteins and peptides by SID-SRM-MS were significantly different between cases and controls (Fig 3A). Although GM and BG were significantly elevated in cases vs controls, the distribution of values was highly skewed with many probable IPA cases having low values that overlapped with controls. We also observed that circulating alpha-1-acid glycoprotein (A1AG1), leucine rich globulin (LRG1), fibrinogen (FIB) -A and -B chains, and alpha antitrypsin (AATC)/ SERPINA1 proteins were elevated in IPA vs matched controls (Fig 3A). By contrast, albumin (ALBU) and apolipoprotein (APO) A1 were reduced in IPA compared to matched controls, as were retinol binding protein (RBP) and APOA2 peptides (Fig 3A). These findings qualitatively reproduced our discovery results, indicating that IPA in leukemia is associated with dysregulation of circulating anti-proteases and coagulation factors. However, because the range of each protein measurement significantly overlapped that of the control population, none of these markers was diagnostic in isolation. We therefore focused on developing a discriminant biomarker panel for patients with a primary underlying diagnosis of leukemia.

### Verification and predictive modeling for IPA

An independent group of 57 patients (43 males, 14 females) with an average age of 52.54 ± 14.4 years with a primary diagnosis of leukemia was tested as the verification cohort: Cases and controls were indistinguishable for age, gender distribution, and neutrophil count (Table 1). Moreover the age and gender distribution of this verification cohort was not statistically different than that of the original discovery cohort. We measured abundance of the candidate marker panel consisting of 8 plasma proteins, 4 peptides and fungal antigens (Fig 3B). Although IL-6 was also observed to be increased in IPA, this marker contributed no independent information and was not further pursued. In this verification cohort, the expression of fungal antigen (GM and BG), host proteins (LRG1, FIBA and AATC), and 3 host peptides from APOA2 were consistently significantly different between cases and controls, in the same relative expression pattern as that observed in the discovery data analysis (cf. Fig 3A and 3B). We also noted that although the differences in A1AG1, ALBU, APOA1, FIBB and A1AT did not achieve statistical significance, the median values showed a similar directional trend as that seen in the discovery cohort. These data constituted the independent verification data set for constructing discriminant classifiers.

To identify the best method for combining the differentially expressed proteins, we evaluated a group of machine learning classifiers for performance in the IPA discovery/qualification data set using 10-fold cross validation. Recognizing that the data were not parametrically distributed, and the performance of machine learning classifiers are highly dependent on the underlying data structures [22, 25], we evaluated the performance of classification and regression trees (CART), random forests (RF), multivariate regression spine (MARS), and generalized pathseeker (GPS) machine learning techniques by their classification accuracy and area under the ROC curve (AUC). Although all classifiers evaluated generated accurate models on the training data set, there were significant differences in their performance on the verification data set (Table 6). Here, the GPS classifier produced the highest overall AUC (0.775) and the smallest decrement in AUC between the qualification (training) and the verification (test) data set (Δ = 0.131). These data indicated that the GPS is the most robust classification approach for the IPA data set.

**Table 6:** Comparison of performance of machine learning classifiers.

PREDICTIVE MODEL	Accuracy	Accuracy	Accuracy	AUC	AUC	AUC
	Train	Test	Δ	Train	Test	Δ
CART	0.941	0.633	0.308	0.941	0.633	0.308
RF	0.821	0.594	0.227	0.891	0.69	0.201
MARS	0.916	0.632	0.284	0.959	0.689	0.27
GPS	0.740	0.557	0.183	0.906	0.775	0.131

Abbreviations: CART, classification, and regression tree; RF, random forest; MARS, multivariate adaptive regression splines; GPS, generalized pathseeker. For each classifier, the area under the ROC curve (AUC), and Δ, the difference in AUC between the training and test data set are shown. Note that the AUC of the RF classifier is lowest, indicating that this classifier will generalize to new data sets.

### Classification of IPA using fungal antigens

To evaluate the classification performance of fungal antigen measurement, we developed GPS-based discriminant classifiers using GM as a continuous variable from the qualification cohort as the training data set and those from the independent verification cohort as the test data set. The GPS classifier using GM alone produced poor accuracy of 0.5 for both the training and test data (Table 7). These data indicate that classifiers using fungal antigens alone are likely to be unreliable.

**Table 7:** Comparison of variable performance of GPS classifiers.

Predictive Variables	Accuracy	Accuracy	AUC	AUC
	Train	Test	Train	Test
GM Only	0.50	0.50	0.699	0.863
Host Proteins Only	0.475	0.519	0.921	0.753
All 14 variables	0.74	0.557	0.906	0.775

For each classifier, the area under the ROC curve (AUC), and Δ, the difference in AUC between the training and test data set are shown. Note that the Δ AUC of the RF classifier for all 14 variables is stable, indicating that this classifier will generalize to new data sets. Accuracy represents case group only.

### Performance of the IPA RF classifier incorporating host response proteins

We next constructed a discriminant classifier using the host response proteins and peptides alone or combined with the fungal polysaccharides in the qualification cohort as the training data set and performance evaluated on the verification data set, as above. Here, the GPS classifier using combined fungal polysaccharides and host response proteins yielded the highest accuracy on the verification data set than either the classifier using GM or host proteins alone (Table 7).

**Fig 4:**
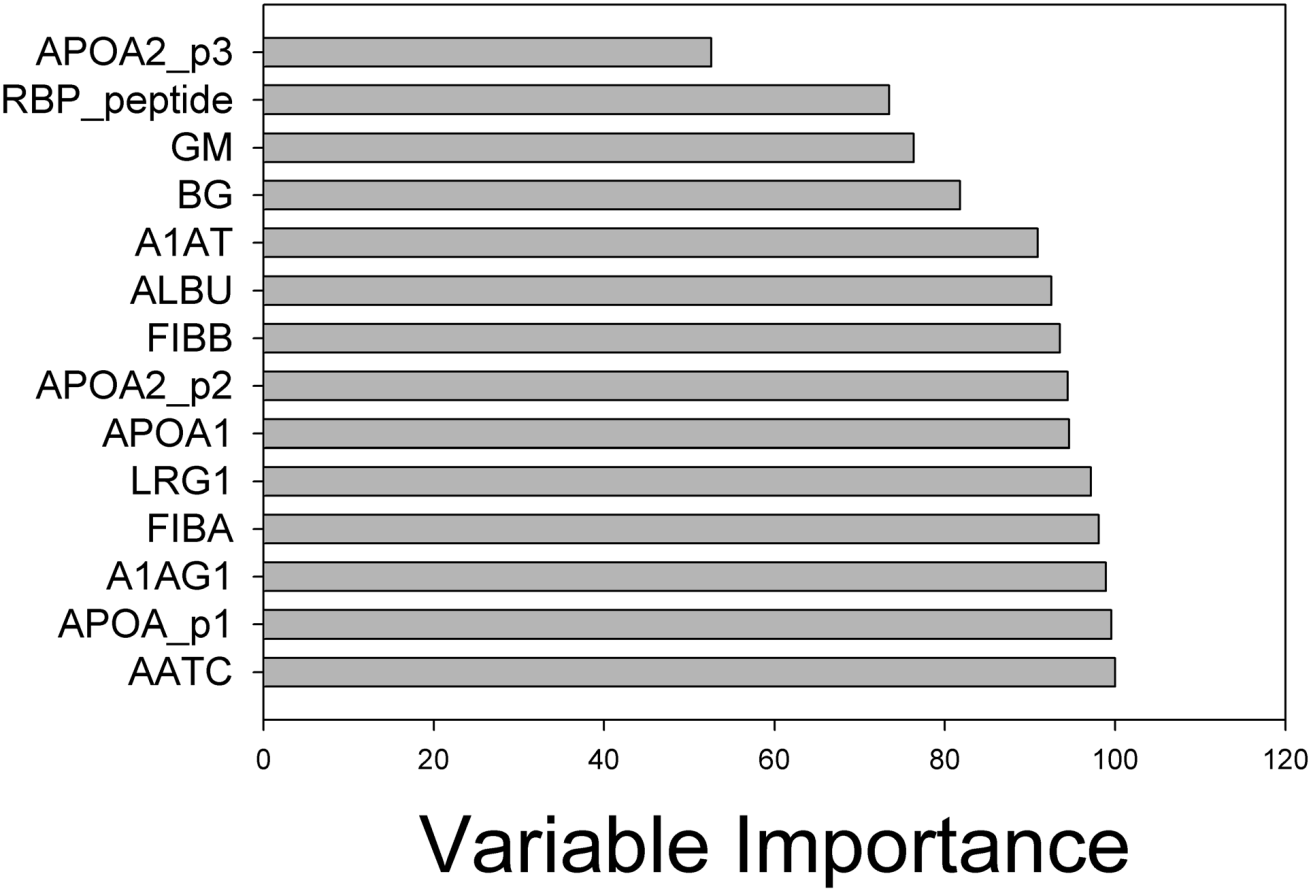
Candidate variable importance of the RF IPA model. Variable importance is a relative measurement from 0–100% that indicates the level that each marker contributes to the performance of the classifier. Note the top three important variables are host response proteins.

**Fig 5:**
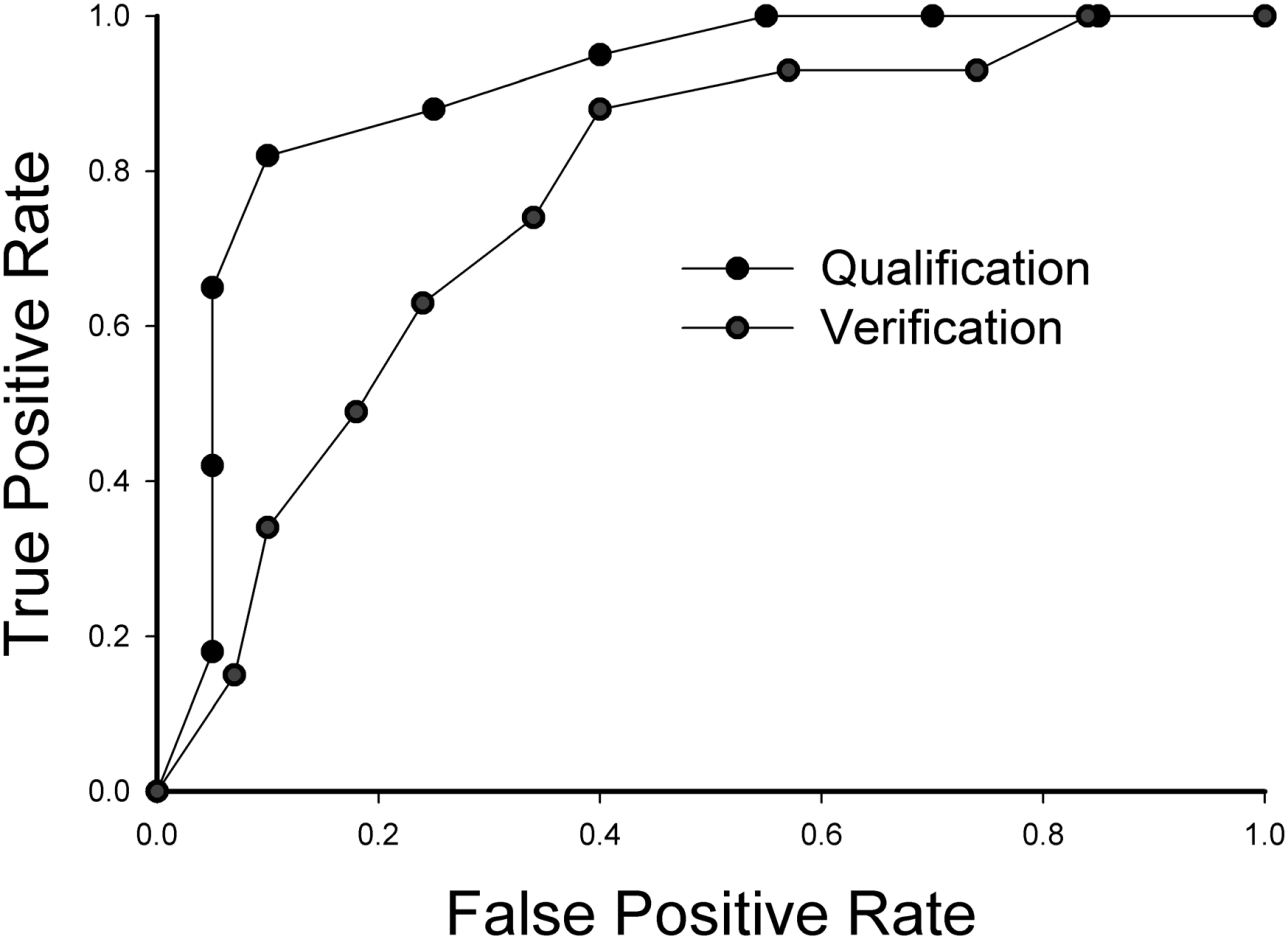
ROC Curve for the RF IPA prediction. ROC for GPS classifier using host response proteins, BAP peptides, and fungal antigens (BD and GM). AUC values for training and test data sets are given in Table 6.

The performance of the GPS classifier using host response proteins combined with fungal polysaccharides for predicting IPA arising during chemotherapy treatment for leukemia was assessed using several approaches. First, we examined the features most important for classifier performance using the “variable importance” measure. Here, 10 host response proteins, the top-most representing APOA1 p1, AATC and ALBU were the most informative features in the classifier, each having variable importance values of >90% (Fig 4). Although BG and GM had significant variable importances of 82% and 76%, respectively, these factors contributed less information than the above-referenced host response proteins, (Fig 4). The performance of the classifier using ROC is shown in Fig 5.

## Discussion

*Aspergillus* is an opportunistic fungal pathogen that accounts for the greatest mortality due to mold pathogen infections of immunocompromised patients, particularly those undergoing chemotherapy for hematological malignancies [26]. In this setting, one of the most important single risk factors is prolonged and profound neutropenia, indicating the primary role of the neutrophil in preventing inhaled conidia from becoming invasive [8]. In neutropenia, early diagnosis of IPA and prompt intervention with anti-fungal therapy improves survival [14, 15]. Despite this recognition and the availability of sensitive ELISAs for fungal cell wall components and polysaccharides, the diagnosis of IPA is clinically imprecise [6, 9, 16]. In this study, we sought to discover, qualify, and verify a panel of protein biomarkers associated with proven and probable IPA. We applied discovery proteomics analyses in plasma samples obtained from well-characterized patients participating in a prospective observational study. A candidate marker panel consisting of 8 plasma host response proteins, 4 abundant protein fragments, a fungal cell wall component (BG), and a fungal polysaccharide (GM) were selected by statistical filtering. Our data suggest that addition of host response markers may be important additions to detect IPA, and will provide opportunities for earlier interventions and interventional clinical trials.

In this study, we approached IPA biomarker discovery in plasma, because this represents an clinically accessible biofluid in which aspergillosis antigens can be found [11]. However, the discovery of biomarkers in plasma samples from humans is a challenging undertaking. Work from the human plasma proteome project has shown that plasma contains high-abundance proteins and tissue leak proteins that span a dynamic range of nearly 12 orders of magnitude [27], including low-abundance proteins circulating in complexes with carriers [28]. Moreover, pathological processes may induce the formation of post-translationally modified proteins and proteolytic fragments that may be pathogenic for tissue-invasive disease, such as IPA. To address these difficulties, we describe here the application of high recovery sample prefractionation strategy to detect both proteins and peptides [18]. The initial denaturation of the plasma prior to rapid SEC fractionation avoids the pitfall of peptide loss through its binding to high-abundance plasma carrier proteins. Moreover, SEC is a nonadsorptive, high recovery prefractionation approach that achieves 95–100% recovery of the input protein. Finally, our development of a quantitative saturation fluorescence labeling approach results in accurate, quantitative 2DE to identify differentially expressed proteins [19, 29]. Here we observe that alterations in isoelectric point and protein mass of abundant proteins important in the complement cascade occurs in a subset of patients with proven and probable IPA that would be otherwise not detected using conventional “bottom-up” analysis of tryptic peptides. These changes in isoelectric points suggest that these proteins are being post-translationally modified during the process of IPA. Further work will be required to identify the modifications and the processes involved. Additionally, we observe that IPA is associated with reduced levels of circulating RBP and APOA2 peptides, suggesting that these factors may be locally consumed during tissue invasion or degradation of extracellular matrix.

The transition from *Aspergillus* colonization into invasive disease is a complex, orchestrated response of the fungus interacting with the host. Stimulated by changes in the microenvironment, inhaled conidia transform into hyphae, where fungal growth results in direct angio-invasion releasing GM and BG into the circulation [6, 7]. Previous work has identified elevated endothelial products including vWF and Factor VIII in of chronic necrotizing bronchopulmonary aspergillosis [24]. Although measured, these factors were not found to be elevated in this cohort. Taken together with our finding that biomarker patterns are different in patients with primary hematological malignancy (leukemia) *vs* lung transplants, and stem cell transplants, informative host response biomarkers of IPA may be highly dependent on the primary underlying disease state. Further quantitative proteomics analysis of probably cases of IPA in lung transplants or stem cell transplants will be required to identify disease-relevant pathways and markers of IPA in these conditions.

Produced as a consequence of hyphal growth, the polysaccharide GM is used clinically for the identification of IPA [9, 11, 12] [30]. Because of its uptake by neutrophils, variations in assay performance by site of infection, previous anti-fungal therapy, or antigen clearance, GM measurement is unreliable as a stand-alone diagnostic of IPA [11]. In this study, although the mean population measurements of GM is increased in cases vs controls, a GPS classifier based on GM alone is unstable and unlikely to generalize. Similarly, BG is a cell wall component present in *Aspergillus* and a wide variety of other pathogenic fungi including *Candida*, *Fusarium*, and *Pneumocystis*. As a diagnostic for IPA, BG measurements may be artifactually influenced by *Candida* colonization, or bacterial infections [31]. We note that BG produces poor performance classifiers in much the same manner as those by GM, and in combination, a classifier built with both GM and BG does not produce a robust AUC for predicting independent test data (not shown). By contrast, classification performance of a panel including these two fungal antigens and host response markers provide greater diagnostic power for leukemic IPA than either host response or fungal antigens alone.

Although the design of this experiment was not to identify pathogenic mechanisms in IPA, the spectrum of 66 proteins that we identified may provide some insights into host response to IPA in leukemic patients. Previous work has shown that the complement cascade represents a crucial first line of innate defense against invasive fungi [32, 33]. Upon contact with plasma, *Aspergillus* activates the classical-, lectin-, and alternative complement pathways that collectively converge on the C3b convertase. Downstream of activated C3b, formation of the terminal membrane attack complex results in pathogen killing [34–36]. We observe that complement factors B, C3, and C4A are differentially expressed by IPA patients and are subjected to post-translational modifications (Table 3). These data suggest that complement consumption is a component of the pathogenesis of leukemic IPA.

Multiple fibrinogen (FIB) isoforms were also identified in our differential analysis, including FIB-A and -B (Table 3). Other studies have shown that fibrinogen specifically binds to *Aspergillus fumigatus* conidia in a dose-dependent manner, appearing to be involved in promoting cell adhesion [37]. It is interesting to us that FIB levels are increased in IPA cases, suggesting their enhanced synthesis in response to fungal dissemination. FIB is induced by IL-6, a cytokine whose expression is enhanced in IPA by our ELISA measurements, perhaps suggesting that an IL-6-FIB pathway is activated in response to angioinvasion in patients with leukemia. Leucine-rich glycoprotein (LRG) is also differentially expressed and an informative component of our biomarker panel. LRG is also involved in cellular adhesion, although its role in promoting fungal host invasion has not been elucidated to our knowledge.

Angio-invasion in IPA is partly dependent on the secretion of extracellular proteinases and ribonucleotoxin [38]. We also note that AATC and alpha-1-acid glycoprotein antiproteases are also differentially expressed. We surmise that induction and consumption of AATC and A1AG are observed in high probability IPA.

Finally, there are several important limitations of this study. One limitation is the low number of subjects in the verification arm indicating that this study will need to be extended to a larger cohort. A second limitation is that our 2DE analysis identified a number of post-translationally modified and proteolyzed peptides for which we could not selectively measure. More advances in understanding the nature of these post-translational modifications and development of methods to detect them in a complex background of plasma may improve the performance of the host response proteins as a classifier. Our study design was not intended to assess specificity of the panel for other invasive fungal diseases. Further work will be required to determine the performance of these markers in patients with other types of invasive fungal diseases.

### Summary and potential applications

IPA is important opportunistic infection affecting immunocompromised patients. Guided by the need to establish an early diagnosis, we have identified, confirmed, and evaluated a multicomponent predictive panel for the presence of IPA in a prospective cohort of immunocompromised patients in a multi-center registry. Two important findings are that host response proteins contribute independent information to that of GM or BG, and that diagnostic host response proteins of IPA are significantly influenced by the primary underlying disease. Our findings may suggest strategies for further refining diagnostics to identify early stages of angio-invasion in IPA arising during intensive chemotherapy for leukemia.

## Supporting Information

S1 Fig Log transformed IL-6 values in case vs control. (PDF)

S1 Table Diagnostic criteria for probable or proven IPA. N, number of subjects. Abbreviations: Asp, aspergillosis; Histo, histopathology; Cyto, cytopathology; NT, not tested.
(PDF)

S2 Table Distribution of subjects with proven/probable IPA and positive GM. (PDF)

S3 Table Cytokine expression. Test Statistics from Wilcoxon ranked sum of log2 transformed data.
(PDF)
