# Supplementary figures and images for: Improved Detection of Invasive Pulmonary Aspergillosis Arising during Leukemia Treatment Using a Panel of Host Response Proteins and Fungal Antigens
Source: PLoS One. 2015 Nov 18;10(11):e0143165. doi: 10.1371/journal.pone.0143165 (PMC4651335; doi:10.1371/journal.pone.0143165)

**S1 Fig. Log transformed IL-6 values in case vs control**

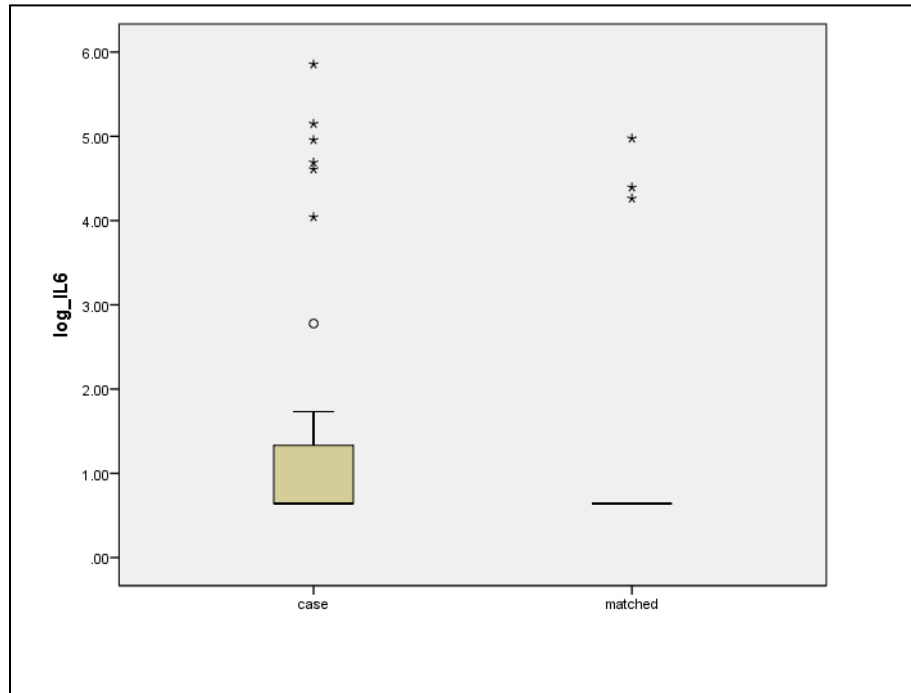

Supplement: S1 Fig — (PDF) [file pone.0143165.s001.pdf]
