# Supplementary material for: Improved Detection of Invasive Pulmonary Aspergillosis Arising during Leukemia Treatment Using a Panel of Host Response Proteins and Fungal Antigens
Source: PLoS One. 2015 Nov 18;10(11):e0143165. doi: 10.1371/journal.pone.0143165 (PMC4651335; doi:10.1371/journal.pone.0143165)
Supplement: S1 Table — N, number of subjects. Abbreviations: Asp, aspergillosis; Histo, histopathology; Cyto, cytopathology; NT, not tested. (PDF) [file pone.0143165.s002.pdf]

S1 Table. Diagnostic criteria for probable or proven IPA. N, number of subjects. Abbreviations: Asp, aspergillosis; Histo, histopathology; Cyto, cytopathology; NT, not tested.

N	Serum GM	BAL GM	Fungal Culture for Asp	Direct Exam	Histo	Cyto
22	Positive	Neg or NT	Neg or NT	Neg or NT	Neg or NT	Neg or NT
1	Positive	Neg or NT	Positive (Sputum)	Neg or NT	Neg or NT	Neg or NT
1	Positive	Neg or NT	Neg or NT	Neg or NT	Neg or NT	Positive
9	Positive	Positive	Neg or NT	Neg or NT	Neg or NT	Neg or NT
2	Positive	Positive	Neg or NT	Neg or NT	Neg or NT	Positive
1	Positive	Positive	Positive	Neg or NT	Neg or NT	Neg or NT
15	Negative	Positive	Neg or NT	Neg or NT	Neg or NT	Neg or NT
2	Negative	Positive	Neg or NT	Neg or NT	Neg or NT	Positive
1	Negative	Positive	Positive (Sputum)	Neg or NT	Neg or NT	Neg or NT
3	Negative	NT	Positive	Neg or NT	Neg or NT	Neg or NT
1	Negative	NT	Neg or NT	Positive	Neg or NT	Positive
2	NT	NT	Positive	NT	NT	NT
