# Supplementary material for: Improved Detection of Invasive Pulmonary Aspergillosis Arising during Leukemia Treatment Using a Panel of Host Response Proteins and Fungal Antigens
Source: PLoS One. 2015 Nov 18;10(11):e0143165. doi: 10.1371/journal.pone.0143165 (PMC4651335; doi:10.1371/journal.pone.0143165)
Supplement: S2 Table — (PDF) [file pone.0143165.s003.pdf]

S2 Table. Distribution of subjects with proven/probable IPA and positive GM

**Serum GM positive**	**36**
**Alone**	22
**With BAL also positive***	12
**With culture also positive ***	2
**With microscopy also positive ***	3
**Serum GM negative**	22
**With BAL GM positive***	18
**With culture positive ***	4
**With microscopy positive***	3
**Serum GM not tested**	2
**With culture positive**	2
***Some may have multiple mycologic criteria**	
