# Supplementary material for: Improved Detection of Invasive Pulmonary Aspergillosis Arising during Leukemia Treatment Using a Panel of Host Response Proteins and Fungal Antigens
Source: PLoS One. 2015 Nov 18;10(11):e0143165. doi: 10.1371/journal.pone.0143165 (PMC4651335; doi:10.1371/journal.pone.0143165)
Supplement: S3 Table — Test Statistics from Wilcoxon ranked sum of log2 transformed data. (PDF) [file pone.0143165.s004.pdf]

S3 Table. Cytokine expression. Test Statistics from Wilcoxon ranked sum of log2 transformed data.

	IL6_matched - IL6	TNFa_matched - TNFa	IL10_matched - IL10	PAI1_matched - PAI1	FACTOR_matched - FACTOR	vWF_matched - vWF
Z	-1.988[b]	-1.342[b]	-.504[b]	-.652[b]	-.697[b]	-1.867[b]
Asymp. Sig. (2-tailed)	.047	.180	.614	.514	.486	.062
